# Qualitative Analysis of Substance Uses Among Wollega University Undergraduate Students, Wollega, Western Ethiopia, 2025: An Exploratory Qualitative Study

**DOI:** 10.1002/hsr2.72678

**Published:** 2026-06-30

**Authors:** Firezer Belay Keno, Eba Abdissa, Adisu Ewunetu, Desalegn Biru, Gelane Gurmu, Lalisa Mekonnen, Desalegn Abebe, Keneni Ephrem, Segni Mulugeta Tafasa, Worku Fikadu, Wabi Tamasgen, Getahun Fetensa

**Affiliations:** ^1^ School of Public Health, Institute of Health Science Wollega University Nekemte Ethiopia; ^2^ Department of Anthropology, Faculty of Social Science and Humanities Wollega University Gimbi Ethiopia; ^3^ Gimbi General Hospital, West Wollega Oromia Ethiopia; ^4^ West Wollega Zonal Health Bureau Oromia Ethiopia; ^5^ German Leprosy and TB Relief Nekemte Ethiopia

**Keywords:** educational reason, environmental related factors, psychological distress, socio‐cultural factors, substance use

## Abstract

**Background and Aims:**

Substance use is a rising public health concern among University students. Nowadays, most university students use substances for different purposes, but it has negative impacts on university students, ranging from social, economic, psychological, and mental problems. This impact, in turn, results in poor academic performance or dropping out of university. There were limited qualitative study designs that explored substance use among university students in Ethiopia, which were used to understand why most students use those substances. So, this study aimed to explore the reasons for substance use among Wollega University undergraduate students, Wollega, western Ethiopia, 2025.

**Methods:**

An exploratory qualitative study design was employed from March 25, 2025 to May 2, 2025. Exponential discriminatory snowball sampling and purposive sampling method were used to sample study population. Semi‐structured in‐depth interview and key informant interview guides in English were created to standardize data gathering by referencing a variety of previously published works. Data were audio‐recorded, transcribed verbatim, and imported into qualitative data analysis software, Open Code version 4.03, for coding and analysis.

**Results:**

Twenty participants were included, with 13 in‐depth interviews and 7 key informant interviews. About six themes emerged about the reasons why the majority of Wollega University students take substances, including: due to sociocultural reasons, educational factors, to alleviate psychological distress, university‐related factors, laws, and environmental considerations.

**Conclusions:**

The study revealed that most of Wollega university students use substances due to socio‐cultural factors, educational reasons, psychological problems, laws, and university‐related factors, and environmental factors. Therefore, given the potential for far‐reaching consequences, immediate and effective prevention and control strategies are urgently needed to address substance use among students.

**Abbreviations**
ASEAN: Association of Southeast Asian Nations
CGPA: cumulative grade point average
e.g.: example
HIV/AIDS: human immunodeficiency virus/acquired immunodeficiency syndrome
IDI: in‐depth interview
PhD: doctor of philosophy
STD: sexually transmitted diseases

## 1 Introduction

Substance use is an important public health problem and a pervasive global issue with varying prevalence and characteristics across different countries among university students [1, 2]. Substances are defined as any substance (except food and water) which, when taken into the body, changes the way the mind (e.g., perception, consciousness, cognition, and mood and emotions) and body work [3, 4]. The effects of substance use on university students have received considerable research attention due to their substantial effects on the young generation. University students use this new system due to changes in the environment and social network as they have enrolled in university.

The percentage of substance users differs from region to region and from country to country. For example, a previous study shows that more than 16.9% of Southeast Asian students use substances at least once. Studies also show that over 2.2% of Asian university students use substances in the last 12 months [5]. The lifetime rate of psychoactive substance use among university students in developing countries was 84.5% [6]. In Kenya, about 48.6% of university students use substances; currently, about 37.9% of university students use substances [7]. In Ethiopia, a previous study demonstrates that the majority of university students use substances ranging from alcohol, tobacco, and khat, marijuana, and cocaine. A study conducted at Adama Science and Technology graduating class implied that the prevalence of current substance use was 55.2%, whereas, the study undertaken at Ambo University shows that about 72.6% of the students use substances [8, 9].

Substance use has considerable negative effects on the users, ranging from hangover or illness, missing class, being a waste of resources like money, to experiencing memory loss [10]. Most university students are more likely to use substances, and are at high risk of engaging in substance use, which has various impacts on their physical and mental health, as well as their socio‐economic and academic achievement [11]. Still another author argues that excessive use of substances can lead to vulnerability to contracting HIV/AIDS and other sexually transmitted diseases (STDs), and multiple psychiatric disorders [12]. Consequently, consuming those substances by the students also results in poor academic performance in class, school dropout, bullying other students, lack of interest in studying, low concentration, and stealing [13].

Scholars argue that university students use substances due to pressure from friends, students' easy access to substances, to get relief from stress, affordability, and family history of substance use were major factors that incite university students to use substances [9, 14]. Other authors argue that the use of substances at university is attributed to personal pleasure, to stay awake, to get acceptance by friends, academic dissatisfaction, and personality problems that range from losing family members and loved ones, and limited recreational areas [10, 11, 15].

However, the strategies have not reduced the number of students taking substances. It is against this background that this paper seeks to identify the factors influencing drug and substance abuse and find out the effects that drugs and substance abuse have on university students [16, 17]. In our study area, specifically at Wollega University, a limited study was conducted on substance use, and some of the studies done were quantitative ones and did not explore why most students use substances; hence, this study tried to explore factors that instigate university students to use substances. We argue that this will fill the gap related to the availability of literature.

## 2 Materials and Methods

### 2.1 Study Area and Period

Wollega University is located in the Oromia Regional State, western Ethiopia. The three campuses are Nekemte, Gimbi, and Shambu. Nekemte Campus is the primary campus in Nekemte city, which is administered by the East Wollega Zone. Gimbi Campus is situated in the West Wollega Zone. Shambu Campus is located in the Horro Guduru Wollega Zone, which is part of the Shambu town administrative zone. When we conducted this research, Wollega University had 119 undergraduate, 44 postgraduate, and 15 PhD active programs across three campuses. The investigation was carried out from March 25 to May 2, 2025.

### 2.2 Study Design

An exploratory qualitative study design was employed to understand why Wollega University students use substances.

### 2.3 Study Population

Populations selected for this study were Wollega University undergraduate students who use substances, student council, hotel waitresses around campus, and university security guards.

### 2.4 Inclusion Criteria

University students who use substances at least for 3 months; university securities, hotel waitresses around campuses, and students' council are included in this study.

### 2.5 Exclusion Criteria

Participants who fulfill the criteria but decline to participate in the study were excluded from the study.

### 2.6 Sample Size and Sampling Technique

#### 2.6.1 Sampling Size Determination

Scholars argue that the sample in qualitative data was determined by data saturation; data saturation is related to the degree to which new data repeats itself in previous data [18]. Therefore, 20 participants were included with 13 in‐depth interviews (IDI) and 7 key informant interviews (KII); the interviews were planned for a qualitative sample size, expecting to reach saturation at this point. In addition, the interviews were undertaken with seven students and one with the student council, two with campus securities and two with hotel waitresses around campus. Participants were chosen as they were users of the substances; others had information on University students' substance use.

#### 2.6.2 Sampling Procedure/Technique

In this research, non‐probability sampling technique was used to select the sample population; from non‐probability sampling techniques, exponential discriminatory snowball sampling was employed in order to identify the substance users; the selected populations were targeted for in‐depth interviews. From non‐probability sampling techniques, the purposive sampling method was used to identify key informants for the interview.

### 2.7 Data Collection

To standardize data collection, semi‐structured IDI and KII guides in English were developed, drawing upon a range of existing literature. To maintain credibility, in‐depth questionnaires were evaluated by qualitative experts from Wollega University in order to improve the validity and reliability of the research findings. The tool was pre‐tested on two participants before actual data collection. The data were collected through face‐to‐face interviews.

### 2.8 Trustworthiness

To maintain credibility, in‐depth questionnaires were evaluated by qualitative experts from Public Health, Anthropology Departments, and Wollega University in order to enhance the accuracy of the research. Besides, so as to reduce the potential bias while collecting, transcribing, coding, and analyzing the data, many scientific methods were used. For instance, during data collection, the questionnaire was distributed among the selected population; an in‐depth interview was employed to enhance the data collected through the questionnaire. Then, the actual data collection was conducted by two health professionals; researchers participated in transcribing, coding, and analyzing the data; information was shared, discussed, and evaluated by the authors; finally, a summary of core points and some confusing ideas was presented to informants.

### 2.9 Data Processing and Analysis

In the data processing and analysis, data were audio‐recorded, transcribed verbatim, and imported into qualitative data analysis software with Open Code version 4.03 for coding and analyzing data. First, the collected data (recordings) were transcribed into text form and then written in Microsoft Word plain text. The second step was importing the text into Open Code. The third step was coding the data; it involves identifying the main feature of the collected data and making notes. Notes can be in the form of codes. Each code corresponds to a particular research question, to which the text was relevant. The next step was generating initial themes (synthesis). The fifth step was reviewing the themes (synthesis); these all helped us to ensure thematic analysis. The next step was naming and defining the themes; these all helped to organize analysis and ensure that not to be confused among different themes and data.

### 2.10 Ethical Consideration

Before the actual data collection process, ethical clearance was obtained from Wollega University Institute of Health Sciences Department of Public Health, and brought to the Wollega University Registrar's Office, Student Council, and responsible bodies to get permission for the study. Confidentiality was ensured and maintained by the principal investigators and data collectors during data collection.

## 3 Result

### 3.1 Socio‐Demographic Characteristics of the Informants

Twenty participants were included; 13 IDIs with students who uses substances and 7 KIIs were dormitories, student council, hotel waitresses around campus, and university security guards who were purposively selected from Wollega University undergraduate students who use substances (Table 1).

**Table 1:** Demographic characteristics of study participants on substance use among Wollega University undergraduate students, 2025.

Types of respondents	Purpose of inclusion	Types of substances used by informants	Sex	Age	Residence prior to joining the university
IDI1	Substance user	Alcohol and cigarette	M	22	Urban
IDI2	Substance user	Khat	M	22	Rural
IDI3	Substance user	Alcohol	M	23	Urban
IDI4	Substance user	Khat	M	24	Rural
IDI5	Substance user	Khat and alcohol	M	23	Urban
IDI6	Substance user	Alcohol, khat, and cigarettes	F	21	Urban
IDI7	Substance user	Alcohol, khat, cigarettes, and marijuana	M	22	Urban
IDI8	Substance user	Alcohol	M	19	Urban
IDI9	Substance user	Alcohol and cigarette	M	21	Urban
IDI10	Substance user	Alcohol	M	21	Urban
IDI11	Substance user	Alcohol	F	20	Urban
IDI12	Substance user	Alcohol and khat	M	21	Rural
IDI13	Substance user	Alcohol, khat, and cigarettes	M	23	Urban
Types of interview	Role	Sex		Age	
KII1	Dormitory	M		35	
KII2	Dormitory	M		28	
KII3	Hotel waitress	F		30	
KII4	Head of security	M		35	
KII5	Student council	F		20	
KII6	Security	M		41	
KII7	Hotel waitress	F		26	

### 3.2 Reasons for Substance Use Among Wollega University Undergraduate Students, Western Ethiopia, 2025

According to the majority of respondents, most students use substances for many reasons; those reasons are categorized into five themes. First, they use them for concerns of socialization, environmental reasons, policy and rules of the campus, educational reasons, and psychological problems (Table 2).

**Table 2:** Themes and sub‐themes on the reasons for substance use among Wollega University undergraduate students, 2025.

Theme	Sub‐theme
Socialization	Peer pressure
	Family history of substance use
	Religious holidays and celebrations
	To relax with friends and strengthen the bond
	Perceiving substance use as sign of modernity
Educational reason	To improve academic performance
Psychological problems	Stress related to the campus environment
	Stress related to personal problems
Laws and university‐related factors	Absence of strict laws on substance use
	Insufficient awareness creation on substance use
	No one assigned a responsible body to follow about substance use
Environmental factors	Substance found on campus
	Substances are easily found around campus
	Absence of recreational area on the campus

### 3.3 Reasons That Instigate Students to Use Substances Throughout University Life

#### 3.3.1 Theme 1: Socio‐Cultural Reasons

As it is indicated from qualitative data analysis, socio‐cultural factors play a major role in instigating the majority of university students to engage in substance use throughout their campus life. Peer pressure, family history of substance use, religious holidays, ceremonies, and festivities are the most commonly identified factors to have caused university students to use substances. Other factors are attributed to viewing substance use as a sign of modernity, viewing them as a sign of enjoyment with friends, creating social bonds with different groups of people, and enhancing relationships with students.

##### 3.3.1.1 Peer Pressure

According to the majority of informants, one of the factors that pressurized Wollega University students to use substances throughout their campus life is attributed to peer pressure; It is mandatory to get adequate information about the campus life and academic issues for freshman students; hence, they must create relationships with senior and junior students who are already indulged in drugs and substance uses in the campus. One of the participants in the interview from the Gimbi campus, IDI7 has explained the influence of the peer as follows:

“I have been using alcohol, khat, cigarettes, and marijuana since I joined university; Unfortunately, I have been assigned with students who have the experience of using substances; from day to day, my dorm mate enticed me to join them; I just see myself as a part of that group.”

##### 3.3.1.2 Family History of Substance Use

Some of the students use substances because they were used by their family before joining Wollega University. Specifically, female students make substances like alcohol with their mom; they prepare them for the rest of their family. IDI6 has explained her experience and link with substance use as follows:

“I started to use alcohol when I was with my family; seeing what my family is doing, I have learnt from them how to make local wine; after that I have become accustomed to drinking it; I drink all alcoholic drinks; however, ever since I have joined campus, I have been accustomed to drinking alcohol in rare cases.”

Another informant also added a similar experience in the following ways:

“First, my family; I mean my father uses khat because he sells it for a living; I started chewing khat when I used to assist him in trading khat; by the way, my father did not allow us to use khat, but my brother and I used to chew it by hiding ourselves from my father.”

(IDI4)

##### 3.3.1.3 To Celebrate Religious Holidays

The interview result shows that students who don't actively use substances often try to use them during some occasions, namely, religious holidays and festivities. Current use of substances by female students has familial and historical connections. At the family level, most female students who are currently using substances to make alcohol at home for holidays before joining Wollega University. As soon as they enrolled in university, they recalled the memory of festivity they used to enjoy during the holiday; on the campuses, they wait for similar occasions to happen. However, on campus, they are not able to get alcohol, which they used to use during holidays, and have turned to using alternative alcohol consumption, such as beer, wine, etc. IDI8 has explained the way students' uses alcohols in the campus as follows:

“The campus compound is almost empty during the night of holidays; most students use substances like alcohol, namely beer, and wine, etc.; most of the users in this campus use them due to peer pressure; probably, students will continue to use them in the future because they are highly addicted to them.”

##### 3.3.1.4 To Relax and Strengthen the Bond With Their Friends

Most of the interviewed students have indicated that they use substances for relaxation and strengthening a bond with other students; just similar to holidays, substance users usually use them during weekends; substance users typically use them during weekends as a part of campus life; mostly, the day out of weekends are working days; students often get the opportunity to invite each other during these weekends; substance users use not only alcohol during the weekends, they also use khat (*Catha edulis*) as additional recreation and relaxation; alcohols usually are used after chewing a khat; consequently, it is argued that substance uses during different occasions have held together chewers' social, economic, and political bonds.

##### 3.3.1.5 Perceiving the Use of Substance as Modernity

The majority of informants argue that they use substances for different purposes throughout their campus lives; first, the use of substances namely khat, cigarettes, and marijuana, is typically perceived as modern person by numerous users; joining a higher institution like university is also seen as a modern person. Because, university is seen as the place where behavioral change occurs; accordingly, to demonstrate behavioral change such as modernity in the university, substance users usually lower their trousers down to the tip of their buttocks, handling a piece of cigarette between their fingers; they also lay a hat on their head with the front face of the hat towards the back.

#### 3.3.2 Theme 2: Educational Reasons

Under this theme, sub‐themes are categorized and condensed into one sub‐theme. The reason why most substance users use substances will be explained in the following subtopics.

##### 3.3.2.1 To Improve Academic Performances

Most of the students who use substances like khat have said that they typically use it to improve their academic performance; for users, khat stimulates, inspires, and motivates them; it removes their dizziness, exhaustion, and overtiredness due to reading many books on campus. IDI2 from natural science has explained the reason why he uses substances as:

“Many students use khat today; they, including me use khat because it lessens our dizziness, exhaustion, and overtiredness and provokes us to be alert, vigilant, and attentive the whole day while reading; without chewing khat I feel dizzy, depressed and worry; I do not always cover all the topics; I usually give up to study easily if I don't use khat.”

A 22‐year‐old, male informant has provided similar views, arguing that:

“Anytime when I use substances like khat, it arouses me to vigilantly concentrate my attention merely on my study; the time I used khat, I can easily master the burden of assignments and exams that are endowed from diverse teachers from different courses”.

#### 3.3.3 Theme 3: For Relieving From Psychological Distress

Under this theme, two subthemes will be analyzed based on interview with informants. First, it discusses the link between substance use and environmental factors; second, it discusses the link between substance uses and personal factors. I start with the link between the link between substance uses and environmental factors throughout students' campus lives.

##### 3.3.3.1 Stress Related to Campus Environment

According majority of informants, substance users use substances such as khat, cigarette, and marijuana because most of campus life needs concentration while studying, to score good results; sometimes when students start to worry excessively about the number of the pages they have to read, they found themselves in the middle of substance use; most of students say that their beginning of khat is unexpected situation; other users of khat are lower academic achiever; many students who use substances are indulged when they have scored lower grades and sets for dismissal from university; owning to lower grades, poor academic performance and worries of dismissal from university are some of the factors that have pressurized them to use substances in the campus. A similar argument is explained by IDI4 as follows:

“I was dismissed from university; I started to use those substances in order to hide my emotions; at that time I was highly over vexed; when I started to use those substances, I felt healthy; I gained my confidence back.”

##### 3.3.3.2 Stress Related to Personal Problems

According to some informants, substance users at Wollega University use substances for their own personal problems. The latter is associated with the death of family members, separation from parents, including their loved ones, and family social, political, and economic problems. One of the informants has described the way he started substance use as follows:

“I started substances after I have separated from my girlfriend; I extremely love her; we had never separated before I joined this university; by the time I missed her I considered myself as if I had died; following separation from her, I was so depressed at that time; the only alternative I have is that finding medicine to my mind; I directly indulged myself in substance use, namely chewing, smoking and drinking; and finally, I have regained my mind as I have begun to use them.”

IDI13

#### 3.3.4 Theme 3.4: Laws and University‐Related Factors

The majority of informants have also said that substance users use substances like khat, drugs, and alcohol owing to the absence of strict laws and inadequate awareness creation in the university on the negative impacts of substance use. Informants have also mentioned that there is no one who is assigned to teach, direct, and guide substance users throughout their university lives. This is also another reason for using substances among many Wollega University undergraduate students.

##### 3.3.4.1 Theme 3.5. Absence of Strict Laws

Each and every one of the informants has asserted that everybody knows substance use is legally not permitted on campus. Nonetheless, the problem is that the law is not strict; if everybody violates the rules and begins to use substances, there is no law that enforces it on campus. Not only students, but also everyone, including those who guard the campus, university communities, and student representatives, as well as law enforcers, use them. Hence, it has become a normal condition on campus. A 24‐year‐old, male informant has described the link between the absence of strict law on campus and accompanying substance use in the following ways:

“There is a law that prevents us not to use any substances in university. Surprisingly, the security guards use substances like tobacco and khat in the university compound but they sometimes prevent us from using those substances pretending they are enforcing the law; sometimes they catch us with some substances, but they return to us; no administrative measure taken to prevent us.”

IDI4

##### 3.3.4.2 Insufficient Awareness Creation on Substance Use

Informants also indicated that insufficient awareness creation on substance use also plays its role in instigating many students to use substances at Wollega University. Currently, the majority of students use substances on campus owing to insufficient awareness creation. In addition, there is no anti‐ substance use club in the campus that gives awareness and gives continuous follow‐up to students who are addicted to substances. One student mentioned a similar argument in the following ways:

“Sometimes, some group comes and creates little awareness as a campaign and disappears. I think it is better if they give detailed awareness; users should receive adequate awareness of the long‐term negative impacts of substance use on students.”

IDI6

##### 3.3.4.3 No One Is Assigned as the Responsible Body to Follow the Use of Substances

The interviewees also asserted that a lot of students at Wollega University use substances, as no one has been assigned as a responsible body to follow up and avert substance users from using them. One of KII1 said that:

“We know the rules that forbid the use of any substances in the campus, but in the campuses students use them; no one is assigned to follow and take appropriate measures against those who violate the rules of the university.”

#### 3.3.5 Theme 5: Environmental Factors

a. Substances found in the campus environment
  Users also contend that environmental factors are responsible for their beginning of substance use; students have planted drugs on the campus; no one knows the color and the type of the leaves of the tree; anyone who sees them on the campus considers them simply a tree naturally grown on the campus. This is amazing and incredible information I have received from IDI4; he has explained during the interview as follows:
  “Most of the substances found around campus areas, bars, and other places. By the way, students who have come from other places, including the Shashamene area, brought the seed of marijuana to the campus; we planted it on the campus; when I was in the main campus we used it, and nobody knows it, and it looks like grass and has smaller leaves and everybody who saw it considers it as a grass but we only know it.”
  Another informant has provided similar information, arguing that
  “We use substances; those substances are mostly found around campus, hotels, bars, shops, and khat shops. In rare cases, I have friends who use ganja; my friends have planted them in campus compound; no one knows the color and advantage of the tree. Nonetheless, it is we who know nature and use them; my friends give me when I ask for them.”
  IDI1
  A student from Gimbi campus explained that
  “There is beer in our campus specifically, the teachers' lounge and this means our teachers test and sometimes students who have a good relationship with the lounge owner use those beers and imagine what this means?”
  IDI5
b. Substances easily found around campuses
  Almost all of participants in the interview have argued that substances like khat, cigarette, and alcohol are found around campuses. On the main campus, some substances like marijuana are grown and nurtured by students. However, in the remaining campuses, namely in Shambu and Gimbi, students have to go downtown to search for marijuana. This is explained by one of IDI7 as,
  “When an institution is established in one area, such as hotels, shops, and other businesses are built around campuses, those substances also appear with them.” This shows that there are links between substance use and the opening of business institutions around the campus.
c. Absence of recreational options on the campus

Participants in the interview also mentioned that the absence of recreational options on the campus environment usually pushes students to use substances by moving far away from the campus; users usually go to town to find out unhealthy recreational options like alcohol, khat, and illegal substances. A 24‐year‐old male has asserted that “there is no way to be out of campuses to search for something that makes me relax in campuse if many recreational options are available on campus.

## 4 Discussion

The purpose of this study is to investigate substance use among students at Wollega University. According to the study, the majority of Wollega University students take drugs for a variety of reasons, including sociocultural, educational, psychological, laws, and academic‐related issues, as well as environmental concerns. According to informants, peer pressure was the most common reason for substance use in this study, which is consistent with findings from studies conducted at two institutions in Kenya, Niger Delta University [19, 20]. Most university students lived in the same neighborhood and shared comparable interests. Classmates, housemates, close acquaintances, and senior students were all regarded as peers on campus [21]. Other relevant factors that the students explored were substance use during certain ceremonies and holidays. According to participants from various institutions, they utilize substances like alcohol on birthdays, celebrations, parties, and holidays; which is consistent with the majority of studies conducted nationwide and internationally [22]. During the holidays, students who have never tasted substance utilize them to relax and celebrate various events.

In particular, parental substance use or the family's financial reliance on the substance trade was found to be among the reasons to start pre‐university substance use. According to studies conducted at Haromaya University and different Ethiopian universities; this prior exposure was predictive of continued substance use during college [20, 21]. This study also matches with the study done at the Bangladesh university, where the use or abuse of any kind of substances by family members appears to influence adolescents' attitude towards substances, which may be because they have easy access to them. Moreover, the habit of use could be developed by seeing their parents or others from childhood in their home [23]. Children have learned and internalized the practices, experiences, behaviors, traditions, norms, customs, and actions that exist within their family, locality, and society with the help and support of their parents. So, parents should caution while using substances in front of their children.

Most of the students who use substances believed that using substances like khat improves their academic performance. This came from the perception that using substances like khat makes them alert the whole night, and without any fatigue, they can study hard. This, in turn, leads to academic performance. This study is in line with the study done in Australia that using substances like stimulants makes the students feel better and study hard [24]. Many students consume a variety of substances in order to achieve good academic results, even though some researchers have suggested that substance use is linked to poor academic performance [25]. In addition, students who use substances have lower academic performance than others due to excessive use of substances leading to school dropout, bullying other students, lack of interest in studying [13, 21, 26]. This implied that using substances can cause memory loss, insomnia, fatigue, and class drop out, and can lead to poor academic performance in general. So, the students and concerned stakeholders should take all responsibilities regarding tackling the problems and promoting students' mental health.

Most of the students use substances due to stress‐related factors, according to this study. It was subcategorized into stress related to campus environment and stress related to personal life. This stress was emerged from environmental factors like dismissal from university, having a fear of low CGPA and fear of being dismissed. The other reason engaged in using substances was stress related to personal life, including the death of family members, separation from their loved ones, and other social, political, and economic problems [9, 15, 27]. Most students misuse substances as a coping mechanism to relieve their stress. They thought using those substances made them feel better and forget their problems, hiding themselves in those substances. Misusing substances may seem to give them immediate comfort or a temporary escape. However, it can lead to more psychological or physical health concerns in the long run [28]. So, the students use positive coping mechanisms when they face problems related to stress.

Insufficient awareness creation on the negative consequences of substance use was another reason explored by Wollega university students to use substances. This study is in line with the study done at Mekelle University, and insufficient awareness results in poor knowledge, attitude, and perception toward the harmful effects of substances use [16]. Awareness of substance abuse involves recognizing the types of substance abuse and their harmful effects, as well as understanding that addiction is characterized by compulsive seeking behavior despite adverse consequences [29]. This, in turn, results in poor academic performance and other health‐related problems among university students. The university should work on this to prevent the problem by considering different preventive measures, including health education on substance use and introducing substance use preventive club on campus environments.

Absence of strict laws was another reason to use substances among Wollega university students. This is in line with the study done at Mekelle University [16]. This is due to no one being assigned to follow the problem and take appropriate measures. So, university administrators should strictly work on this and should assign a responsible committee who follow the problem. The other reason was that there were no recreational options in the campus environment, and students were looking for those options instead. Lastly, substances were easily accessible by the students on the campus environment and around campus area and that's why most of the students consume them. This study is in line with the study done at Haromaya University, Ambo University, and Addis Ababa University [9, 15, 21]. It is simple to get substances due to easy availability in universities. So, university administrators and administrators around the campus environment should work together to tackle the problems.

## 5 Conclusions and Recommendations

The study revealed that most of Wollega University students use substances due to multiple reasons, including socio‐cultural factors, educational reasons, psychological problems, laws and university‐related factors, and lastly environmental factors. Therefore, addressing substance use among students requires immediate and proactive prevention and control measures to mitigate further harm.

## Author Contributions

**Firezer Belay Keno:** conceptualization, investigation, writing – original draft, methodology, writing – review and editing, validation, formal analysis, software, supervision, resources, project administration, visualization, data curation. **Eba Abdissa:** conceptualization, writing – original draft, writing – review and editing, formal analysis, software, supervision. **Adisu Ewunetu:** writing – original draft, writing – review and editing, formal analysis, software, methodology, supervision. **Desalegn Biru:** conceptualization, investigation, writing – review and editing, writing – original draft, resources, supervision, project administration, methodology. **Gelane Gurmu:** conceptualization, methodology, formal analysis, supervision, writing – original draft. **Lalisa Mekonnen:** writing – original draft, writing – review and editing, project administration, software, methodology, investigation. **Desalegn Abebe:** resources, supervision, writing – review and editing, validation. **Keneni Ephrem:** conceptualization, writing – original draft, methodology, writing – review and editing, formal analysis, supervision, resources, investigation. **Segni Mulugeta Tafasa:** writing – original draft, conceptualization, writing – review and editing, methodology, project administration, resources. **Worku Fikadu:** conceptualization, writing – original draft, visualization, formal analysis, project administration, supervision, data curation. **Wabi Tamasgen:** writing – review and editing, writing – original draft, investigation, project administration, formal analysis, supervision, resources, methodology. **Getahun Fetensa:** writing – original draft, writing – review and editing, methodology, conceptualization, formal analysis, project administration, supervision.

## Funding

The authors have nothing to report.

## Ethics Statement

Before the actual data collection process, an ethical clearance was obtained from Wollega University Institute of Health Sciences Ethics Review Committee (IHS REC) on March 18, 2025 with Reference number IHS REC/03/465/2025. Then, it is brought to Wollega University registrar's office, student council, and responsible bodies to get permission for the study. Verbal informed consent was taken from informants, and confidentiality was ensured and maintained by the principal investigators and data collectors during the data collection process.

## Conflicts of Interest

The authors declare no conflicts of interest.

## Transparency Statement

The lead author, Firezer Belay Keno, affirms that this manuscript is an honest, accurate, and transparent account of the study being reported; that no important aspects of the study have been omitted; and that any discrepancies from the study as planned (and, if relevant, registered) have been explained.

## Data Availability

All the data and materials are available with the authors.
